# A comprehensive assessment of the transcriptome of cork oak (*Quercus suber*) through EST sequencing

**DOI:** 10.1186/1471-2164-15-371

**Published:** 2014-05-15

**Authors:** José B Pereira-Leal, Isabel A Abreu, Cláudia S Alabaça, Maria Helena Almeida, Paulo Almeida, Tânia Almeida, Maria Isabel Amorim, Susana Araújo, Herlânder Azevedo, Aleix Badia, Dora Batista, Andreas Bohn, Tiago Capote, Isabel Carrasquinho, Inês Chaves, Ana Cristina Coelho, Maria Manuela Ribeiro Costa, Rita Costa, Alfredo Cravador, Conceição Egas, Carlos Faro, Ana M Fortes, Ana S Fortunato, Maria João Gaspar, Sónia Gonçalves, José Graça, Marília Horta, Vera Inácio, José M Leitão, Teresa Lino-Neto, Liliana Marum, José Matos, Diogo Mendonça, Andreia Miguel, Célia M Miguel, Leonor Morais-Cecílio, Isabel Neves, Filomena Nóbrega, Maria Margarida Oliveira, Rute Oliveira, Maria Salomé Pais, Jorge A Paiva, Octávio S Paulo, Miguel Pinheiro, João AP Raimundo, José C Ramalho, Ana I Ribeiro, Teresa Ribeiro, Margarida Rocheta, Ana Isabel Rodrigues, José C Rodrigues, Nelson JM Saibo, Tatiana E Santo, Ana Margarida Santos, Paula Sá-Pereira, Mónica Sebastiana, Fernanda Simões, Rómulo S Sobral, Rui Tavares, Rita Teixeira, Carolina Varela, Maria Manuela Veloso, Cândido PP Ricardo

**Affiliations:** Instituto Gulbenkian de Ciência, Rua da Quinta Grande 6, Oeiras, 2780-156 Portugal; Instituto de Tecnologia Química e Biológica, Universidade Nova de Lisboa, Genomics of Plant Stress Lab, Av. da República, Oeiras, 2780-157 Portugal; Instituto de Biologia Experimental e Tecnológica, Genomics of Plant Stress Lab, Apartado 12, Oeiras, 2781-901 Portugal; Laboratory of Genomics and Genetic Improvement, BioFIG, FCT, Universidade do Algarve, E.8, Campus de Gambelas, Faro, 8300 Portugal; Centro Estudos Florestais (CEF), Instituto Superior de Agronomia, Universidade de Lisboa, Tapada da Ajuda, Lisboa, 1349-017 Portugal; Centro de Biotecnologia Agrícola e Agro-Alimentar do Alentejo (CEBAL)/Instituto Politécnico de Beja (IPBeja), Beja, 7801-908 Portugal; Centre for Research in Ceramics & Composite Materials (CICECO), Universidade de Aveiro, Campus Universitário de Santiago, Aveiro, 3810-193 Portugal; Faculdade de Ciências, Universidade do Porto, Rua do Campo Alegre, s/n, FC4, Porto, 4169-007 Portugal; Instituto de Biologia Experimental e Tecnológica, Plant Cell Biotecnology Lab, Apartado 12, Oeiras, 2781-901 Portugal; Instituto de Tecnologia Química e Biológica, Universidade Nova de Lisboa, Plant Cell Biotecnology Lab, Av. da República, Oeiras, 2780-157 Portugal; Instituto de Investigação Científica Tropical (IICT), BIOTROP/Veterinária e Zootecnia, R. da Junqueira, 86 - 1, Lisboa, 1300-344 Portugal; Centre for Biodiversity, Functional & Integrative Genomics (BioFIG), Plant Functional Biology Centre, Universidade do Minho, Campus de Gualtar, Braga, 4710-057 Portugal; Instituto de Tecnologia Química e Biológica, Universidade Nova de Lisboa, Systems Biodynamics Lab, Av. da República, 2780-157 Oeiras, Portugal; Instituto de Biologia Experimental e Tecnológica, Systems Biodynamics Lab, Apartado 12, Oeiras, 2781-901 Portugal; Centro de Investigação das Ferrugens do Cafeeiro/BioTrop, Instituto de Investigação Científica Tropical, Quinta do Marquês, Oeiras, 2784-505 Portugal; INIAV- Instituto Nacional de Investigação Agrária e Veterinária, IP, Quinta do Marquês, Oeiras, 2780-159 Portugal; Instituto de Tecnologia Química e Biológica, Universidade Nova de Lisboa, Plant Biochemistry Lab, Av. da República, Oeiras, 2780-157 Portugal; Instituto de Biologia Experimental e Tecnológica, Plant Biochemistry Lab, Apartado 12, Oeiras, 2781-901 Portugal; Instituto de Tecnologia Química e Biológica, Universidade Nova de Lisboa, Forest Biotech Lab, Av. da República, Oeiras, 2780-157 Portugal; Instituto de Biologia Experimental e Tecnológica, Forest Biotech Lab, Apartado 12, Oeiras, 2781-901 Portugal; Centro de Electrónica, Optoelectrónica e Telecomunicações (CEOT), Universidade do Algarve, Campus de Gambelas, Faro, 8005-139 Portugal; Institute for Biotechnology and Bioengineering - Centre of Genomics and Biotechnology (IBB-CGB), Plant and Animal Genomic Group, Universidade do Algarve - Campus de Gambelas, Faro, 8005-139 Portugal; Biocant, Parque Tecnológico de Cantanhede, Cantanhede, 3060 - 197 Portugal; Centre for Biodiversity, Functional & Integrative Genomics (BioFIG), Faculdade de Ciências da Universidade de Lisboa, Lisboa, 1749-016 Portugal; Unidade de Ecofisiologia, Bioquímica e Biotecnologia Vegetal/BioTrop, Instituto de Investigação Científica Tropical, Quinta do Marquês, Av. da República, Oeiras, 2784-505 Portugal; Departamento Genética e Biotecnologia, Univ. Trás-os-Monte e Alto Douro, Vila Real, 5001-801 Portugal; CEF, ISA Technical University Lisbon, Tapada da Ajuda, Lisboa, 1349-017 Portugal; Centro Botânica Aplicada Agricultura (CBAA), Instituto Superior de Agronomia, Universidade Técnica de Lisboa, Tapada da Ajuda, Lisboa, 1349-017 Portugal; Centre for Biodiversity, Functional & Integrative Genomics (BioFIG), Plant Systems Biology Lab, Faculdade de Ciências da Universidade de Lisboa, Lisboa, 1749-016 Portugal; Instituto de Investigação Científica Tropical (IICT), BIOTROP/Florestas e dos Produtos Florestais, Tapada da Ajuda, Lisboa, 1349-017 Portugal; Centro de Biologia Ambiental, Faculdade de Ciências da Universidade de Lisboa, Campo Grande, Lisboa, 1749-016 Portugal; CIBIO, Centro de Investigação em Biodiversidade e Recursos Genéticos, Universidade do Porto, Campus Agrário de Vairão, Vairão, 4485-661 Portugal

## Abstract

**Background:**

Cork oak (*Quercus suber*) is one of the rare trees with the ability to produce cork, a material widely used to make wine bottle stoppers, flooring and insulation materials, among many other uses. The molecular mechanisms of cork formation are still poorly understood, in great part due to the difficulty in studying a species with a long life-cycle and for which there is scarce molecular/genomic information. Cork oak forests are of great ecological importance and represent a major economic and social resource in Southern Europe and Northern Africa. However, global warming is threatening the cork oak forests by imposing thermal, hydric and many types of novel biotic stresses. Despite the economic and social value of the *Q. suber* species, few genomic resources have been developed, useful for biotechnological applications and improved forest management.

**Results:**

We generated in excess of 7 million sequence reads, by pyrosequencing 21 normalized cDNA libraries derived from multiple *Q. suber* tissues and organs, developmental stages and physiological conditions. We deployed a stringent sequence processing and assembly pipeline that resulted in the identification of ~159,000 unigenes. These were annotated according to their similarity to known plant genes, to known Interpro domains, GO classes and E.C. numbers. The phylogenetic extent of this ESTs set was investigated, and we found that cork oak revealed a significant new gene space that is not covered by other model species or EST sequencing projects. The raw data, as well as the full annotated assembly, are now available to the community in a dedicated web portal at http://www.corkoakdb.org.

**Conclusions:**

This genomic resource represents the first trancriptome study in a cork producing species. It can be explored to develop new tools and approaches to understand stress responses and developmental processes in forest trees, as well as the molecular cascades underlying cork differentiation and disease response.

## Background

Oaks (*Quercus* spp.) are important trees of the Northern hemisphere. In Europe they form highly valuable widespread forests. Together with chestnut and beech, oaks belong to the Fagaceae, and are probably the best-known genus of the family. The evergreen cork oak (*Q. suber*) grows in the Western Mediterranean Basin, having as natural range Algeria, France, Italy, Morocco, Portugal, Spain and Tunisia, where it is managed under low-density anthropogenic open woodland forests. *Quercus* spp. are important for conservation of soil and water, biodiversity, natural landscape and climate, and for production of highly valuable materials, thus having high ecological, social and economic value.

*Quercus suber* shares with *Phellodendron amurense* (Amur cork tree) and *Q. variabilis* (Chinese cork oak) the odd ability of producing a continuous and renewable out-bark of cork, although only *Q. suber* cork has the fine physical and chemical properties for a highly profitable industrial use.

Portugal owns the credits of the world leading position on cork oak forest area (740,000 ha out of the world 2,200,000 ha), cork production (60% of the world exported cork volume), and cork processing (74% of world processed cork). In Portugal, in the past, oaks used to dominate the native forests but their area has rapidly decreased as a result of human activity. Still, cork oak forests are accounting for about 26% of the Portuguese forest [1].

However, cork oak (*Q. suber*) and holm oak (*Q. ilex ssp. rotundifolia*) decline reported in the Iberian Peninsula over the last 20 years has caused death of numerous trees, threatening the rural economy in this part of Europe [2–5]. It has been predicted that oak diseases in Europe could become more severe and expand to the North and East within the next few hundred years [6].

Nowadays, this species faces many other threats, such as drought, extreme temperature and pests, leading to a marked decline of cork oak stands, possibly related to the repeated successions of extremely dry and hot years with a significant reduction of springtime precipitation [7].

The relevance of *Q. suber* and the scarce information available on its genetics, biochemistry and physiology [8–14] fully justifies the generation of transcriptomics data that will allow a new insight on cork oak biology and genetics. These data are fundamental for designing selection programs and understanding the plant adaptation processes to both biotic and abiotic factors, plant’s plasticity, ecophysiological interactions, interspecific hybridization and gene flow.

For a species that has neither its genome sequenced, nor a physical map available, the information obtained from expressed sequence tags (ESTs) is a practical means for gene discovery and a way to start elucidating its physiology and functional genome. When this project started (in 2010) there were less than 300 ESTs available for *Q. suber*. Recently, this number has increased to almost 7,000 (http://www.ncbi.nlm.nih.gov/dbEST/dbEST_summary.html).

Other oak species have also been subjected to transcriptomic studies, namely two European white oak species (*Q. petraea*, sessile oak, and *Q. robur*, English oak) [15, 16], two American oak species (*Q. alba*, white oak, and *Q. rubra*, red oak) (reviewed in [17]). Ueno et al. [15] generated 222,671 non-redundant sequences (including alternative transcripts) from multiple cDNA libraries prepared from *Q. petraea* and *Q. robur*, which is a relevant resource for genomic studies and identification of genes of adaptive significance. In 2011, the same team produced another useful tool, a BAC library, for genome analysis in *Q. robur*[18]. Another important tool to develop a physical map for a Fagaceae species was based on the work of Durand and co-workers [19], who produced a total of 256 oak EST-SSRs that were assigned to bins and their map position was further validated by linkage mapping (http://www.fagaceae.org). More recently, [16] generated the larger-to-date set of reads from the transcriptome of an oak species (*Q. robur*), combining 454 and Illumina sequencing.

Within a national initiative, Portugal organized a consortium to study cork oak ESTs (COEC – Cork oak ESTs Consortium, http://coec.fc.ul.pt/), where 12 projects were designed to obtain a deeper understanding of *Q. suber* functional genomics. Developmental aspects (gametophytes, fruit and embryo development, acorn germination, bud sprouting, vascular and leaf development), as well as cork formation and quality, and abiotic (oxidative stress, drought, heat, cold and salinity) and biotic interactions (including symbiosis and pathogenesis) were followed by 20 teams from all over the country. Two of these projects were fully dedicated to the bio-informatics analysis of the generated data and development of bioinformatics platforms, one of them further focusing on polymorphism detection and validation.

This paper presents the experiments conducted for large-scale sequencing of 21 cDNA libraries and construction of a cork oak transcriptome database containing 159,000 unigenes. Presently, this database constitutes one of the largest genomic resources available for oaks and was structured to accommodate future data on genomics and physiology of woody species. The tools that were generated are crucial to study cork oak biology and diversity, and to understand gene regulation and adaptation to a changing environment. Future developments will make possible the early detection of traits of interest. This initiative will contribute to genomic research in cork oak and the Fagaceae family, paving the way for further studies.

## Results and discussion

### Sequencing

We have constructed 21 libraries from *Q. suber* as described in Table 1. The libraries were constructed from total RNA extracted from multiple tissues, developmental stages and stress conditions. Libraries were normalized by the Duplex-Specific Nuclease-technology [20], with the aim of increasing gene-space coverage and sequenced in a 454 GS-FLX with Titanium Chemistry (Roche). A total of 7,445,712 reads were produced, ranging from 40 to 587 bp, with an average length ranging between 185 and 310 bp (Table 2). An initial pre-processing step to remove contaminants, low quality sequences and short sequences resulted in a reduction to nearly 5 million nuclear reads (4,968,463), with average lengths ranging between 209 and 321 bp (Table 2). Our approach resulted in a higher number and comparable read length as compared to other multi-library projects [Moser:2005ju; Ueno:2010bv; ONeil:2010bk; [21]].

**Table 1:** Tissues and conditions used to produce the RNA libraries

cDNAlibrary	Library description
L-1	Phloem (adult trees)
L-2	Xylem (adult trees)
L-3	Abiotic stress: control (leaves)
L-4	Abiotic stress: cold (leaves)
L-5	Abiotic stress: heat (leaves)
L-6	Seed germination
L-7	Female flowers
L-8	Male flowers
L-9	Embryos from fruits at 4 developmental stages
L-10	Whole fruits at 7 developmental stages
L-11	Biotic Stress: roots (germinated acorns) infected by *Phytophthora cinnamomi*.
L-12	Biotic Stress: roots (thin white roots from 18-month-old plants) infected by *Phytophthora cinnamomi*.
L-13	Mycorrhizal symbiosis (roots).
L-14	Annual stems from cork producing Quercus suber x cerris hybrid trees
L-15	Annual stems from cork non-producing Quercus suber x cerris hybrid trees
L-16	Bud sprouting (bud phases 1 and 2).
L-17	Bud sprouting (bud phases 3 and 4).
L-18	Abiotic Stress: drought, salt and oxidative stresses (roots and shoots)
L-19	Leaves (from 8 locations for polymorphism detection)
L-20	High quality cork
L-21	Low quality cork

All libraries were normalized.

**Table 2:** Sequencing statistics

	Raw reads		Processed reads		Individual assemblies		
Library	#	<l>	#	<l>	# total	Contigs	Singlets
L-1	392152	200.2	216861	232.3	30220	26693	3527
L-2	315360	203.0	208162	237.6	23962	21499	2463
L-3	182571	193.6	118708	209.1	16399	15272	1127
L-4	215084	195.7	147735	210.8	19573	18060	1513
L-5	153898	185.2	97870	203.0	14372	13255	1117
L-6	371060	286.7	279793	304.5	32700	27735	4965
L-7	346435	235.1	216309	253.7	30694	28179	2515
L-8	393501	248.9	285776	264.2	33550	29758	3792
L-9	524852	295.0	433762	307.9	48799	37357	11442
L-10	570370	308.3	449849	321.8	50522	39471	11051
L-11	220568	273.4	149645	294.3	18215	17186	1029
L-12	104517	281.2	73958	298.3	8442	8188	254
L-13	743576	248.8	411035	263.7	42318	38830	3488
L-14	413925	271.2	323372	278.6	38794	34102	4692
L-15	401170	261.0	321153	269.2	38359	33447	4912
L-16	320673	259.2	190983	277.7	21694	19607	2087
L-17	350843	262.0	203567	282.3	23857	21989	1868
L-18	774553	254.5	506642	268.6	46983	41086	5897
L-19	650604	272.3	333283	288.9	37926	29543	8383

**Figure 1:**
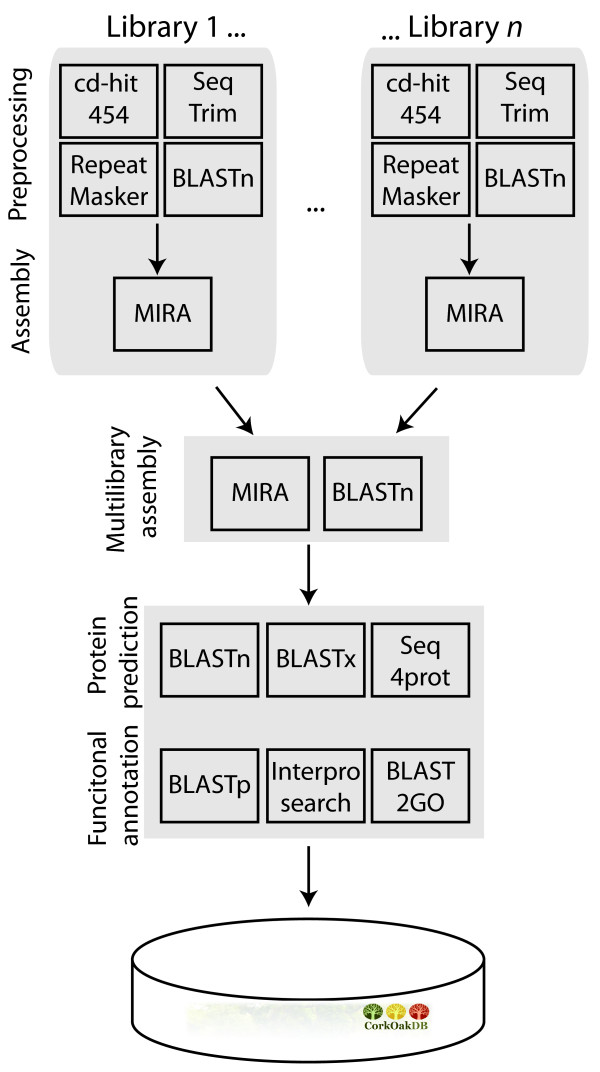
**Schematic representation of the bioinformatics pipeline, indicating the software used at each step.**

Processed Reads represents the number of nuclear sequences after the pre-processing (Figure 1). # stands for number, <l > for average length.

### Assembly

**Figure 2:**
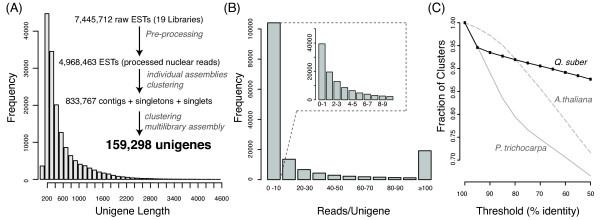
**Assembly and predicted peptide statistics. (A)** Unigene length distribution after multi-library assembly. There are 12 additional unigenes longer than 4600 bases, not shown on the plot, with the longest one being 9189 bases. **(B)** Unigene coverage (reads per unigene). **(C)** Serial clustering of predicted proteins based on the cork oak unigenes, and of the predicted proteins from the genomes of two model plant species.

A stringent assembly pipeline was implemented and is summarized in Figure 1. The assembly methodology is described in the Materials and Methods section, consisting of two stages: first each library was assembled individually, and secondly all assembled libraries were further assembled (assembly of assemblies). The choice of this two-step protocol lied in the asynchronous nature of the libraries being sequenced, and the need to deal with future libraries that are expected to be generated for other conditions and stress types. The choice of parameters in our protocol maximized the number of contigs and their length (in MIRA -‐AL:egp = no:mrs = 85 reduces gap penalties and permits longer matches; -‐AS:mrpc = 1 allows for single read contings, thus increasing the number of contigs), was extensively validated, and is described in greater detail in a companion paper (*in preparation*). We opted for *de novo* assembly, as the lack of a closely related species with a completely sequenced genome resulted in poor assembly (not shown). The assembly statistics for each library are shown in Table 2. A total of 577,852 putative unigenes was achieved, including 501,257 contigs and 76,122 singlets. Each library produced from 8,442 up to 50,522 putative unigenes. These were all subjected to one additional assembly step (see Material and Methods section), which reduced the number of putative unigenes to approximately 159,298 unigenes. The final unigene length distribution is shown in Figure 2A. An average unigene length of 148.5 bp was found, which is smaller than those obtained in another oak using a combination the same sequencing platform with Sanger sequencing [15, 16] (see Table 3). A BlastP of all the unigenes the NR database finds Plant best hits in 97.3% of the cases, with the remaining being hits to other species that are likely contaminations not removed by our pipeline. A plot with the species distribution of these non-plant species is found on CorkOakDB.org.

**Table 3:** Assembly metrics of this project compared with those of two large oak transcriptome sequencing projects

	***Q. suber*** (this study)	***Q. petraea/Q. robur*** [15]	***Q. robur*** [16]
Sequencing platform	454	454 + Sanger	454 + Illumina
Libraries	21	14 (454) + 20 (Sanger)	16 (454) + 8 (Illumina)
Total reads	7,445,712	1,578,192 (454) + 145,827 (Sanger)	821,534 (454) + 255,237,702 (Illumina)
Contigs & single reads	159,298	222,671	65,712
mean length	148.5	235.8	1003

### Coverage and depth

The large number of libraries used, together with the choice of a two-step assembly, resulted in a high redundancy. Most of the nearly 5 million filtered ESTs were assembled into a large number of unigenes (~159 K). We obtained an average coverage depth of 3.9 (number of times each nucleotide was sequenced), with a maximum depth of 429 (25% percentile = 1; 75% percentile = 5). This is higher than other recent tree EST projects using the same sequencing platform (e.g. [22]), likely due to the extensive number of libraries sequenced in this project, prepared from multiple tissues, developmental stages and stress conditions. After the two rounds of assembly, 61,687 high quality reads remained unassembled and were treated as singletons. Thus, 65% of our unigenes derive from contigs, higher than other recent comparable projects (see Table nine in [15]).

In the absence of a complete genome sequence, it is impossible to know the true coverage of the cork oak gene space offered by this project. However, when we queried the proteomes of *Arabidopsis thaliana* and *Populus trichocarpa* using BLASTp to determine the potential number of unique genes detected, using a cut off of e < 10^-5,^ we found that 65% of cork oak unigenes hit 23,482 out of 27,379 predicted proteins in *A. thaliana (85%)*, and 30,318 out of 45,555 in *P. trichocarpa* (67%) [23]. These numbers represent a rough estimate of the upper (85%) and lower (67%) boundaries one can expect from the *Q. suber* transcriptome coverage. This figure doesn’t change significantly if we use a more lenient cut off of e < 10^-2^, where we hit 24,093 (79%) and 30,719 (67%), respectively. A high degree of redundancy in our unigenes is suggested, as multiple unigenes hit the same target genes in either species. The remaining 55,921 unigenes cannot find any hit in either *A. thaliana* or *P. trichocarpa*, representing about 35% of the cork oak transcriptome. These include small unigenes that would not achieve significance in BLASTp comparisons (see Figure 2A), as well as potential novel genes not present in these two genomes. This number could be eventually overestimated, if we consider some under-assembly in our libraries.

We performed a serial clustering at increasing levels of identity in order to evaluate the degree of redundancy in our assembly (Figure 2C). We found that at the protein level, there was a sharp decrease in the number of clusters at 95% identity, indicating that approximately 8000 predicted peptides show a high identity between each other, comparable to that found in other oak species [15]. This could indicate a recent event of polyploidization giving rise to many highly similar genes. Alternatively, and probably most likely, this could be accounted by the high genetic diversity among the multiple unrelated trees used to prepare the libraries [9]. Sequencing errors not fully resolved due to the relatively low coverage of many unigenes could also be responsible for this result. In the first scenario our decision to filter off redundancies at the cDNA level at 98% could have been excessive, leading to the underestimation of the predicted number of unigenes. In contrast, the second and third scenarios would suggest that 95% is insufficient and we are overestimating the number of unigenes that may be closer to 151,000. We do not have enough data to favour any of these scenarios, in particular because all three may co-exist. We have thus chosen the 98% cDNA clustering as a conservative parameter that we hope does not over-cluster paralogues. With future data accumulation, it will be easier to fuse unigenes than to resolve incorrectly clustered paralogues.

### Functional annotation

**Figure 3:**
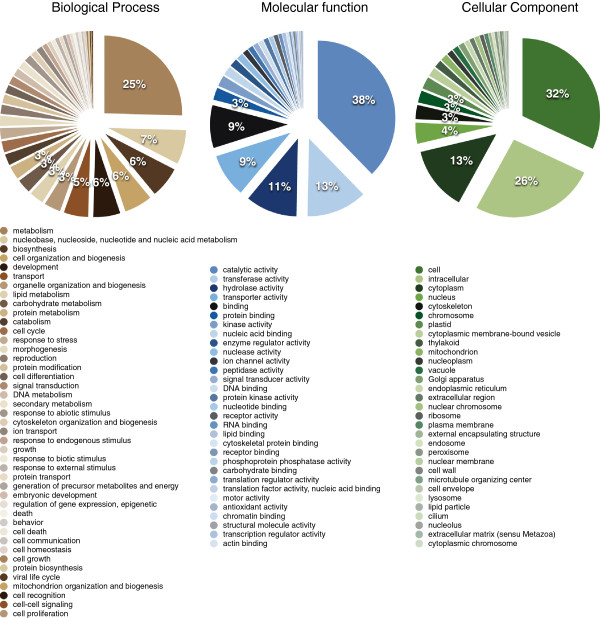
**Gene Ontology classification of nuclear unigenes.** Classification was performed using CateGOrizer, counting single occurrences and the Generic GO Slim [25]. Percentages are shown down to 3% only, and the functional classes are ordered by frequency.

We mapped the cork oak unigenes to the functional classes defined in Gene Ontology (GO) [24]. We had 73,766 sequences mapped to at least one GO term and the unigenes covered a total of 2,273 different GO terms. Each unigene mapped to 3.66 terms on average. The vast majority of terms is present at low frequency, with a few functional classes dominating. The Biological process “Metabolism” was the most frequent, with other metabolic categories in the top five categories - metabolism related categories cover 68% of the terms assigned (Figure 3). Consistently, enzyme functions dominate the Molecular Functions (“Catalytic activity”, “Transferase activity”, “Hydrolase activity”) (Figure 3). These are in contrast with the combined ESTs of two other oaks, *Q. petraea* and *Q. robur*, where the classes Transport (Biological Process) and Nucleotide Binding (Molecular Function) dominate [15]. Note, however, that this difference may simply lie in the fact that in that study non-normalized libraries were used, resulting in under-representation of lowly expressed genes. Furthermore, this difference may also lie in the fact that in that study, nuclear and organelle transcriptomes were, to the best of our knowledge, assembled together, while we removed both chloroplast and mitochondrial sequences from our assembly. This is supported by the observation that in the GO Cellular Component classification, the “Plastid” class is the most frequent in the *Q. petraea*/*Q. robur* ESTs, while in the cork oak, intracellular classes dominate (“Cell”, “Intracellular”, “Cytoplasm”, etc.) (Figure 3).

**Figure 4:**
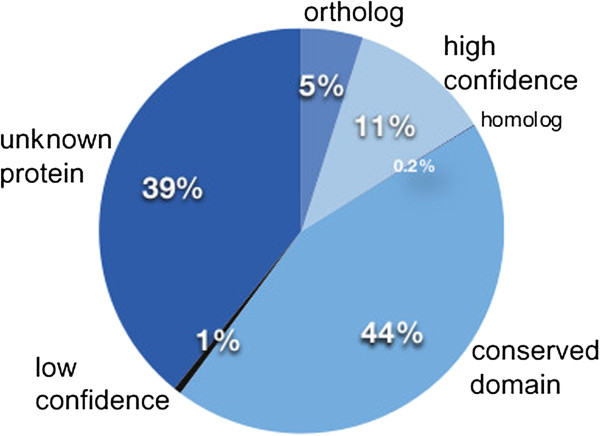
**Distribution of annotation classes in the cork oak translated unigenes.**

We used a simple and conservative scheme for gene naming of the cork oak unigenes. Besides its accession number (see below for details), we gave it an unigene name based on its similarity to proteins in *A. thaliana* and *P. trichocarpa* (Table 4). We observed that for nearly 40% of the unigenes we could not assign a clear annotation at cut off of e < 10^-5^ (Figure 4), consistent with the number of unigenes that are not similar to any gene in other model plants. Conversely, we could identify conserved domains in 44% of the unigenes, and could establish clear homology relationships to an additional 16% of the unigenes, in a total of 60% unigenes with clear functional assignments in GO.

**Table 4:** Unigene naming criteria are as follows

Method		Assignment
***BDBH***		Ortholog
***BLASTp search***		
Alignment length	identity	
> 85%	> 35%	High confidence
> 70%	> 25%	Homolog
< 70%	> 30%	Conserved domain
< 70%	< 30%	Low confidence

If a gene is bi-directional best hit (BDBH) of X in *A. thaliana* (or *P. trichocarpa*), we term it ortholog of X; if it is similar to X in *A. thaliana* (or *P. trichocarpa*) using BLASTp and it aligns in 85% of its length with more than 35% identity, we term it a High confidence X in *Q. suber*, etc.

**Figure 5:**
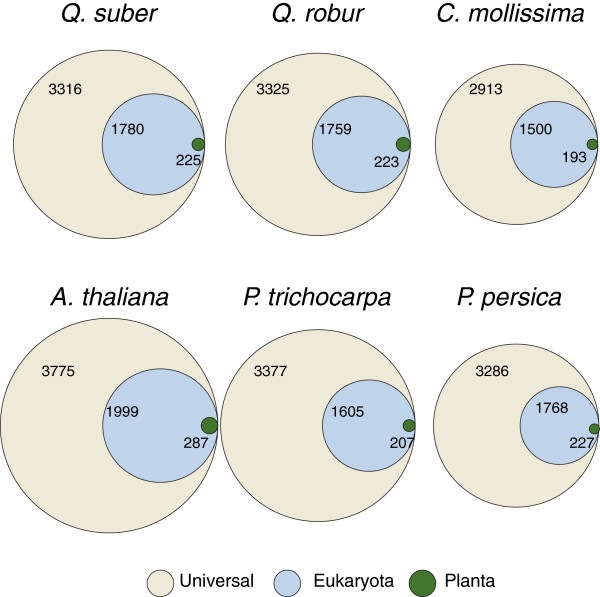
**Unique Interpro domains assigned to the** ***Q. suber*** **unigenes and two other transcriptomes for** ***Q. robur*** **and** ***Castanea mollissima*** **, as well as for species with completely sequenced genomes** ***A. thaliana, P. trichocarpa*** **and** ***P. persica*** **.**

We were able to map Interpro domains to 108,341 unigenes (68%). Nearly half of the domains were widespread in evolution, being present in both Eukaryota and Bacteria (Figure 5). The other half was dominated by general Eukaryotic domains and less than 10% of the domains were plant specific. These results are comparable to those reported for the complete genomes of *A. thaliana, P. trichocarpa* and *P. persica* genomes, as well as to those of the transcriptomes of the closely related *Quercus robur* and *Castanea mollissima* which are also depicted in Figure 5.

### Evolution

**Figure 6:**
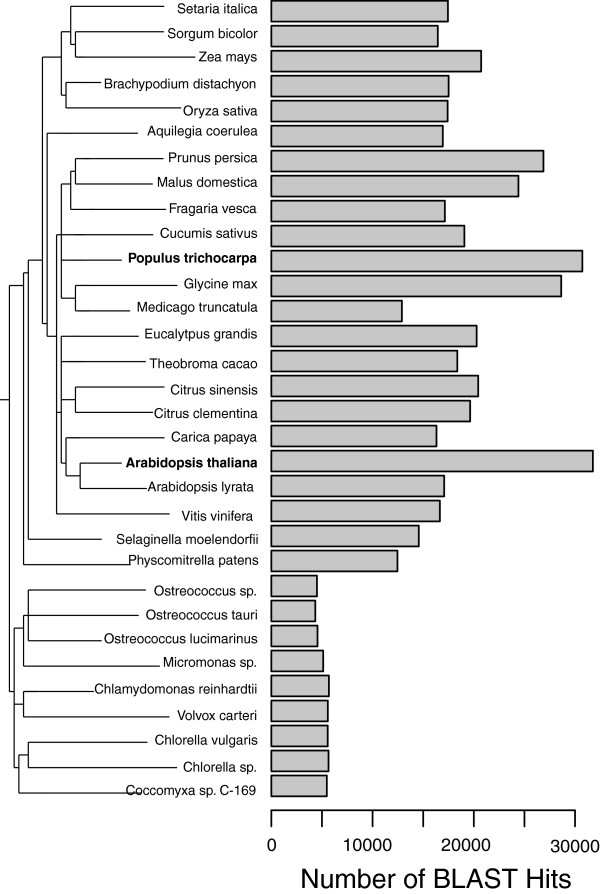
**Number of the cork oak’s predicted peptides unique BLAST hits in other plant genomes.**

We compared the gene content of the cork oak, as estimated by our EST sequencing project, with that of 31 completely sequenced plant genomes. We used BLASTp at e < 10^-5^ and also at the permissive cut off of e < 10^-2^ to determine how many predicted proteins in those species are similar to at least one cork oak unigene. The results of this analysis are shown in Figure 6, indicating a broad concordance with the generic taxonomic/evolutionary distance of the species. This result does not change when we use a more permissive cut off of e < 10^-2^ (not shown).

**Figure 7:**
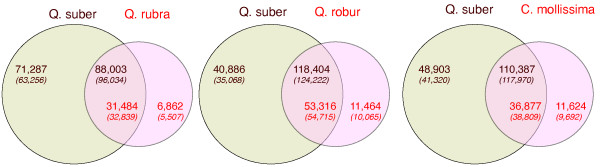
**Overlap between the cork oak unigenes (brown) and the unigenes of the red oak, English oak and Chinese chestnut.** Numbers represent homologues defined at a e < 10^-5^ cut off, and in parentheses at e < 10^-2^.

We compared the unigenes derived from the cork oak with those of the red oak (*Q. rubra*), the pedunculate Oak (*Q. robur* - also known as English or French oak) and the Chinese chestnut (*Castanea mollissima*). For this comparison, the data from the Fagaceae Genome Web was used, for *Q. rubra* and *C. mollissima* which include multiple tissues also sequenced using the 454 pyrosequencing platform (http://www.fagaceae.org/node/87455 and http://www.fagaceae.org/node/181796/, respectively), and the data for *Q. robur*, which included 454 and Illumina generated sequences*,* and was obtained from http://www.ufz.de/trophinoak/index.php?de=31205[16, 26]. We used our own assembly pipeline on these sequences to ensure that no additional differences were introduced on methodological grounds. The comparison is shown in Figure 7. The total number of distinct unigenes is higher in the cork oak project, probably reflecting the higher number of tissues and conditions sampled in our libraries, as well as incomplete assembly due to library biases and genetic heterogeneity of the samples. We verified that between 77% and 82% of the unigenes from those species are similar to at least one unigene in the cork oak, as expected from evolutionarily close species. The remaining 18% - 23% of the unigenes of the red and english oaks and chestnut tree are likely species-specific, but may also be partially accounted by an incomplete coverage of the *Q. suber*. The large number of cork oak unigenes that does not find a hit in the other transcriptomes (30% - 44% at e < 10^-5^) does however suggest that, most likely, this is not a major factor. This cork-oak-specific set represents a mixture of small reads that fail to attain statistical significance (*e.g.* from incomplete assembly), as well as a putative set of cork oak-specific genes. Note that when we compare *Q. suber* with a completely sequenced genome of the *Prunus persica*, 94% of the *P. persica* genes find a hit in *Q. suber*, further suggesting that incomplete coverage of the gene space was probably not a major problem of our project.

### Database and interface

**Figure 8:**
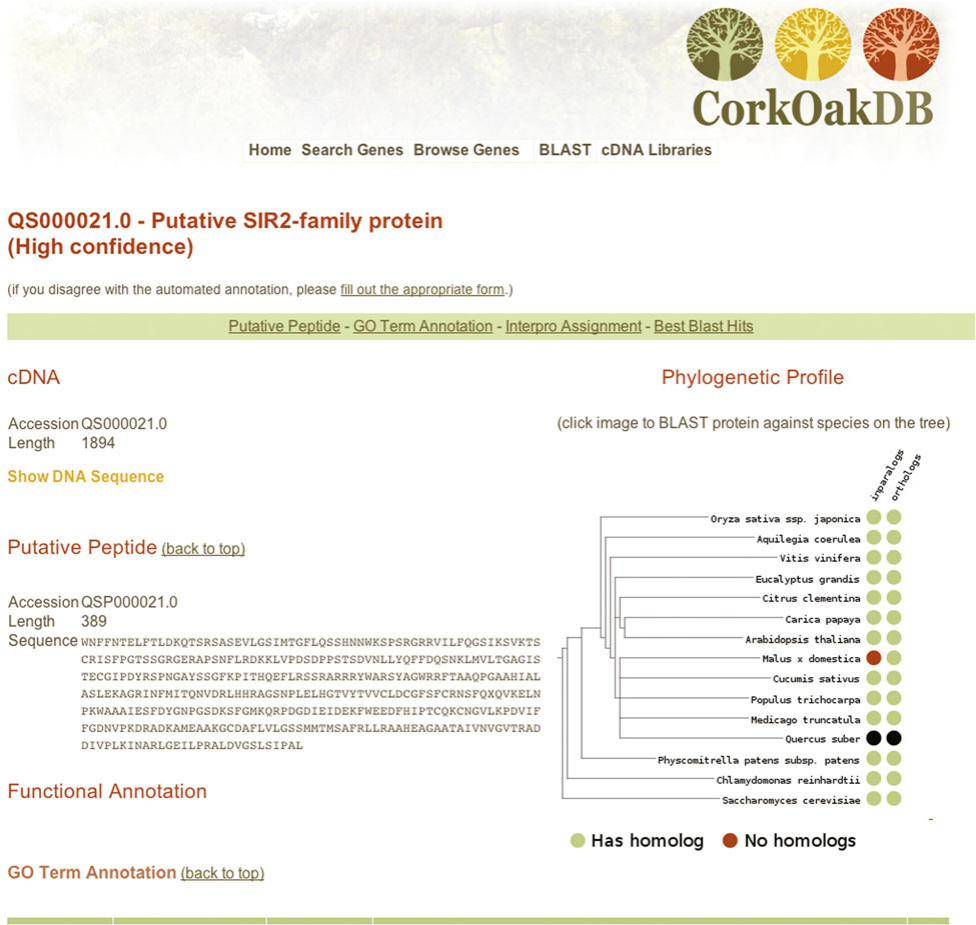
**CorkOakdb.org.** Screenshot of the top part of the gene view.

To support the assembly and annotation pipeline we have a data warehouse system that records the data and metadata associated with each step of the pipeline. This is described in a companion paper (*in preparation*). From this warehouse we generated a public portal as a community resource for cork oak genomics, which is found at http://www.corkoakdb.org. The assembled genes, the proteins they encode, and the functional annotations are made accessible through a web interface, partially shown in Figure 8. The gene view features sequence data, cDNA and protein, as well as plots of base-by-base coverage information for the unigene. Users are shown pre-computed phylogenetic profiles against other plants according to two distinct methods, the bi-directional best BLAST hit and the inparanoid, two standard methods to identify orthologs and paralogues [27]. The gene view further includes functional annotations, namely GO annotations, Interpro domain assignments, KEGG pathways and best BLAST hits against general and plant-specific databases. Genes of interest can be discovered by searching specific fields or by running a nucleotide or protein BLAST search against the Cork Oak database.

## Conclusions

We have developed the first large-scale library for the cork oak, an important economic resource in Southern Europe and North of Africa. We carried out a preliminary analysis of its gene content and functional annotation, and built a public platform for data sharing. Nineteen different libraries were sequenced, covering genes expressed in multiple tissues, developmental stages and stress conditions. Our results suggest that we covered a large fraction of the cork oak gene space. Many of its unigenes are dissimilar to any other plant genes. These likely represent incomplete assemblies due to library biases, but may also include several true cork-oak specific genes, which once identified will represent a promising avenue to understand the molecular basis of the response leading to cork formation. We believe that this sequencing effort will enable the community to explore the molecular basis of the cork oak physiology, as well as its responses to the multiple abiotic and biotic challenges that the cork oak forest is currently experiencing.

## Methods

### Samples, collection and preparation

Within this initiative, in order to guarantee high transcript coverage and to increase gene diversity, total RNA was isolated from *Quercus suber* biological samples obtained from different organs and tissues at varying developmental stages (roots, leaves, buds, flowers, fruits, phellogen, vascular tissue, good and bad quality cork), as well as from plants that had been exposed to infection with *Phytophthora cinnamomi*, symbiosis with *Pisolithus tinctorius* mycorrhizal fungus and different abiotic stresses (cold, heat, drought, salinity and oxidative stress). Furthermore, total RNA was also isolated, at two distinct dates (May and September), from annual shoots of 30 years old Quercus suber x cerris hybrid trees that either produce or don’t produce cork, in order to cover different developmental stages of the phellogen meristem. No approval or licenses were required for sample collection. In each library, plant material from half-siblings (e.g. abiotic and biotic stress libraries) or from several unrelated trees was used. All the plant material used was from Portuguese trees except for those trees used to detect polymorphism, which were from different Mediterranean countries [28]. The detailed conditions applied in each situation are described in http://www.corkoakdb.org/libraries. The full set of libraries is described in Table 1.

### cDNA preparation, library normalization and pyrosequencing

Total RNA from each tissue/condition was used as the source of starting material for cDNA synthesis and production of normalized cDNA libraries intended for 454 sequencing. Briefly, the total RNA quality was verified on Agilent 2100 Bioanalyzer with the RNA 6000 Pico kit (Agilent Technologies, Waldbronn, Germany) and the quantity assessed by fluorimetry with the Quant-iT RiboGreen RNA kit (Invitrogen, CA, USA). A fraction of 1–2 μg of total RNA was used for cDNA synthesis with the MINT cDNA synthesis kit (Evrogen, Moscow, Russia), a strategy based on the SMART double-stranded cDNA synthesis methodology using a modified template-switching approach that allows the introduction of known adapter sequences to both ends of the first-strand cDNA. Amplified cDNA was then normalized with TRIMMER cDNA Normalization kit (Evrogen, Moscow, Russia) using the Duplex-Specific Nuclease-technology [20, 29].

Normalized cDNA was quantified by fluorescence and sequenced in 454 GS FLX Titanium according to the standard manufacturer’s instructions (Roche-454 Life Sciences, Brandford, CT, USA) at Biocant (Cantanhede, Portugal).

### Sequence processing and assembly

The implemented sequence analysis strategy included an initial pre-processing stage, performed on each library, where contaminant, low quality, redundant and repeat-full sequences were removed and each library assembled. This was followed by a multilibrary assembly (described below, and summarized in Figure 1). Initially, each read, respective quality scores and ancillary information, were extracted from the sequencing machine output (.sff), using open source software sff_extract (http://bioinf.comav.upv.es/sff_extract/). Reads of each sample were selected using a Python pipeline that screens the reads for primer sequences, classifying them by sample origin and allocating them in different files. For each sample we generated a file with the sequences (.fasta) and the corresponding file with the quality scores (.qual). At this stage we removed adaptors and reads smaller than 40 bp. Thereafter, artificial duplicates associated with pyrosequencing were removed using cd-hit-454 [30] at a threshold of 98%, and Seq-trim [31] was used to remove small sequences (length < 100 bp) or sequences with low quality (QV > 20, quality window = 10), as well as poly-A or poly-T tails, and adaptors.

In the following step, contaminant sequences were removed. For this, a database of possible types of contaminants was prepared (ContaminantsDB - see supplementary material for details) and queried with the *Q. suber* reads using BLASTn (5, -E 3 -e 1e-09 -q -5 -b 1 -G 3). Reads that found a match in this database, were subsequently blasted against a database of plant proteins (PlantDB - see supplementary material for details) using the same parameters as before. If the hit (match) e-value in ContaminantsDB was smaller than hit (match) e-value in Plant DB, the read was considered as a contaminant and removed from the pipeline. The remaining reads continued in the pipeline to be screened for repetitive elements, using the program RepeatMasker 3.2.9 (http://www.repeatmasker.org) against PlantRepeatsDB [32]. Whenever sequences were masked in more than 90% of their length they were discarded.

The final step of the preprocessing stage was the classification of all the trimmed reads into potential mitochondrial, chloroplastidial or nuclear sequences. For this, a BLASTn (-e = 0.001) was first performed against a database containing coding region sequences from complete plant mitochondrial genomes (from *Arabidopsis thaliana*, *Medicago truncatula* and *Populus tricocharpa*). The sequences that presented a hit were considered potential mitochondrial sequences and were kept in a FASTA file reserved for this organelle sequences. A similar process was then applied against a database of coding region sequences of plant complete plastidial genomes (same organisms).

### Assembly

We chose MIRA 3.2.0 [33] to assemble the resulting sequences, as this has been shown to have higher coverage than other assemblers [34]. For each library, we obtained contigs and singletons with the following parameters: --job = denovo, est, accurate, 454; --GE:not = 20; --SK:not = 20; 454_SETTINGS -LR:mxti = no, -CL:qc = no:cpat = no:mbc = yes, --AL:egp = no:mrs = 85, -OUT:sssip = yes, -AS:mrpc = 1. Following this step, all the contigs and singlets resulting from the assembly of each library were then clustered to remove redundancy using CD-HiT [35], and the resulting non-redundant sequence collection was re-assembled using the same parameters as before. The resulting sequences were considered to be Unigenes, and at this point they were given an unigene accession number. Libraries L20 and L21 were not used in the analysis presented in this manuscript, but are available in the full assembly on the CorkOakDB.

### Protein prediction

In order to be able to translate the nucleotide sequences to protein sequences, the pipeline first performs a Blast search (blastx) against a RNA database [36], to remove non-protein coding unigenes. It then queries all Viridiplantae protein sequences existing in the Uniprot database [37]. The program Prot4EST [38] then takes the outputs of these BLAST searches and translates the sequences into putative peptide sequences. Those unigenes without significant hits are translated using the program ESTscan [39], and for the remaining untranslated sequences, the longest ORF of the 6 frames is selected.

### Sequence naming

In order to assign names to the genes/proteins found, putative peptides were used to query, using BLASTp at a cut off of e < 10^-5^, a database of Uniprot sequences from *A. thaliana* and *P. tricocharpa*. Whenever a putative peptide does not have a hit, it is considered “Predicted hypothetical protein”. If a similar hit is detected, then the protein name is assigned to the putative peptide in *Q. suber* together with a label that describes the level of confidence of the annotation (see Table 4).

### Functional annotation

In order to obtain domains and functional sites of putative peptides, an Interpro search was executed [40]. The Interpro database [41] integrates different classification methods based on amino-acid patterns and profiles, protein family fingerprints, protein sequences and structural domains, as well as functional information. The Interpro database 28.0 was downloaded and searches were run locally. Afterwards, a BLAST (BLASTp) search against non-redundant protein database was executed and results entered the program Blast2GO [42]. We used the pipeline version of the B2G called B2g4pipe, obtaining GO-terms and E.C. Numbers. The same pipeline was used to assign Interpro domains for the transcriptomes analysed in Figure 5.

### Database implementation

A MySQL relational database was deployed, using the InnoDB engine to allow rollback of transactions in case of failure. This was essential, given the progressive nature of the data loading. Every EST sequence was stored in the database, and as each step of the pipeline was ran, the results were added to the corresponding tables, up to the functional annotation of assembled unigenes, as well as metadata related to the EST libraries. Some intermediate output data, such as large FASTA and XML files, were kept on the file system. The web interface is powered by a Python application built on Django (an open source web framework), HTML/CSS and Javascript. KEGG data is displayed using the KEGG SOAP API.

### Accession numbers and unigene naming

Accession numbers on the corkoakDB have the following format QS_000000, for unigenes, and QS_P_000000 for putative peptides. Whenever the sequences are putative mitochondrial or potential chloroplast sequences they start with QSm or QSc, respectively.

### Evolutionary analysis

Comparisons to other organisms were made using predicted proteomes obtained from the superfamily database [43] release 1.75. We used BLASTp for the comparisons, always filtering for low complexity regions and using the cut offs indicated in the text. We used the standard NCBI’s taxonomic tree as a reference for Figure 6. Red oak libraries were obtained from the Fagaceae genomics web (http://www.fagaceae.org/node/87455) and processed using our own pipeline, resulting in 38,346 predicted unigenes. We then used BLASTp with a cut off at e = 0.01 to determine how many unigenes from the cork oak were similar to at least one unigene in the red oak.

### Availability of supporting data

All sequenced ESTs were submitted to the sequence read archive (http://www.ncbi.nlm.nih.gov/sra) with the accession number ERP001762, and accession name “Cork Oak”.
